# Symptomatic Patent Foramen Ovale with Hemidiaphragm Paralysis

**DOI:** 10.1155/2017/9848696

**Published:** 2017-10-16

**Authors:** Hussain Ibrahim, Adnan Khan, Shawn P. Nishi, Ken Fujise, Syed Gilani

**Affiliations:** University of Texas Medical Branch, 301 University Boulevard, 5.106 John Sealy Annex, Galveston, TX 77555-0553, USA

## Abstract

Dyspnea accounts for more than one-fourth of the hospital admissions from Emergency Department. Chronic conditions such as Chronic Obstructive Pulmonary Disease, Congestive Heart Failure, and Asthma are being common etiologies. Less common etiologies include conditions such as valvular heart disease, pulmonary embolism, and right-to-left shunt (RLS) from patent foramen ovale (PFO). PFO is present in estimated 20–30% of the population, mostly a benign condition. RLS via PFO usually occurs when right atrium pressure exceeds left atrium pressure. RLS can also occur in absence of higher right atrium pressure. We report one such case that highlights the importance of high clinical suspicion, thorough evaluation, and percutaneous closure of the PFO leading to significant improvement in the symptoms.

## 1. Introduction

In evaluation of a patient with a chief complaint of dyspnea, a potential pitfall would be to neglect rarer etiologies in favor of more common causes. Dyspnea accounts for 28.6% of the admissions to the hospital from Emergency Department (ED), common etiologies being chronic conditions such as Asthma, Heart Failure, and Chronic Obstructive Pulmonary Disease (COPD) [1]. In light of the number of patients visiting the ED with dyspnea-related complaints, it would be easy to overlook the less common cause of dyspnea. This can impact patient outcomes. Less common causes of dyspnea including valvular heart disease, pulmonary embolism, and intracardiac shunt are curable if the proper diagnosis is made in a timely fashion. We present one such case of treatable dyspnea due to patent foramen ovale (PFO) related right-to-left shunt (RLS) in setting of right hemidiaphragm paralysis. Our review of literature revealed only eleven cases with RLS via PFO in presence of right hemidiaphragm (Table 1) [2–10].

## 2. Case Presentation

**Figure 1:**
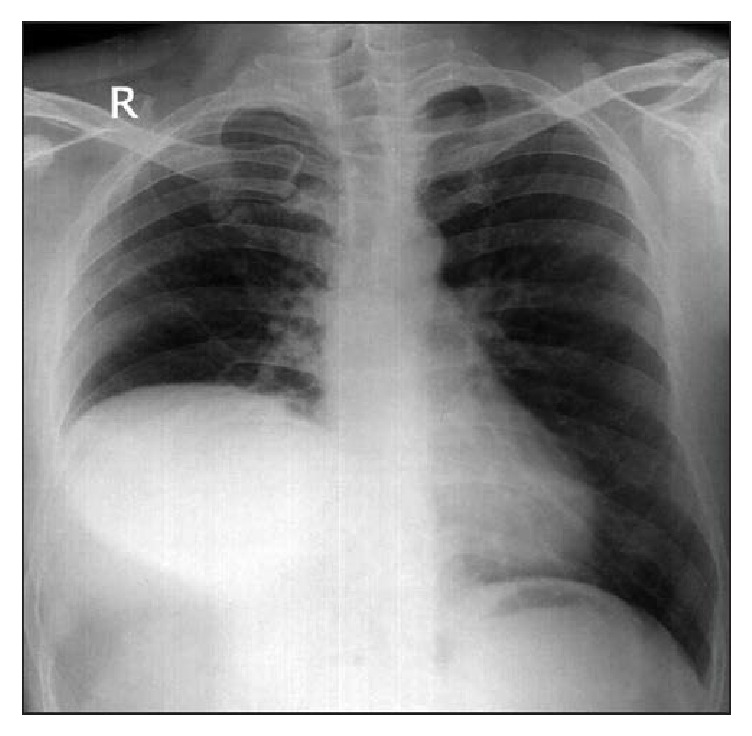
Chest X-ray with right hemidiaphragm paralysis.

59-year-old female with past medical history of long standing primary hypertension, chronic kidney disease Stage III, and Diabetes Mellitus type II started experiencing gradual worsening shortness of breath (SOB) after self-limiting bout of viral pneumonia three months priorly. Her CXR showed right hemidiaphragm elevation that was confirmed with a sniff test (Figure 1).

Detailed pulmonary evaluation for continued SOB also showed moderate COPD and elevated Alveolar-arterial (A-a) gradient on pulmonary function test. Ventilation-Perfusion scan did not show any evidence of pulmonary embolism. Sleep study with apnea-hypopnea index (AHI) of 110 per hour confirmed severe obstructive sleep apnea (OSA) and sleep efficiency of 63.4%.

**Figure 2:**
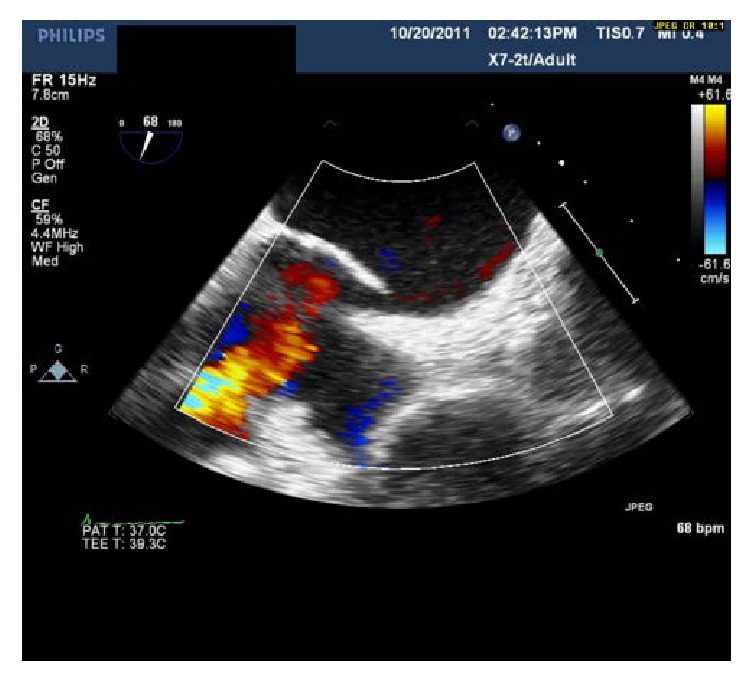
Transesophageal echocardiogram with Doppler demonstrating right-to-left interatrial shunting through the patent foramen ovale.

She was started on continuous positive airway pressure (CPAP) for OSA and on 4 liters/minute (L/min) oxygen via nasal cannula (NC) for continued hypoxia. As part of workup for headaches and hemidiaphragm paralysis, MRI of the brain showed intracranial aneurysm that required craniotomy and clipping. Postoperatively, patient was difficult to extubate due to continued hypoxemia. Therefore, transthoracic echocardiogram (TTE) was performed that showed right-to-left intracardiac shunt on saline bubble study. Transesophageal echocardiogram confirmed a PFO with continuous RLS, normal right ventricle, and right atrium size (Figure 2). Over the next few months, patient required multiple hospitalizations for repeat episodes of SOB presenting to the hospital with oxygen saturations in low 80% despite using continuous 4 L/min oxygen via NC therefore requiring high flow oxygen and continuous positive airway pressure (CPAP) to stabilize her. Acute exacerbation episodes were treated with CPAP and diuresis, and these measures would improve the dyspnea but did not alleviate. Due to persistent hypoxia and lifestyle limiting dyspnea despite oxygen supplementation patient was referred for PFO closure. As part of evaluation for hypoxia and PFO, patient underwent right and left heart catheterization (RHC and LHC) that showed normal coronaries, normal pulmonary pressure and resistance, normal LV filling pressure, and femoral artery O2 saturation of 87% on room air. After heart team discussion including pulmonologist, percutaneous PFO closure was recommended. Patient underwent successful percutaneous PFO closure using 30 mm Amplatzer Cribriform Septal Occluder (ST Jude Medical). She had immediate improvement in her SOB after PFO closure. She was taken off continuous oxygen after 6-minute walk test prior to discharge. Furosemide requirement reduced from 40 mg oral twice daily to 40 mg oral once daily after PFO closure. Her status is at five years after PFO closure with no hospitalization related to SOB. She is currently NYHA Class II, walks > 300 yards, and performs all the activities of daily life without any limitations.

## 3. Discussion

In patients with refractory SOB and hypoxia that fails to resolve with oxygen therapy, a physiologic or anatomic RLS should be considered as part of the differential diagnosis. Workup should include echocardiogram with a bubble study and right heart catheterization. Statistically, the most common cause of an interatrial communication is a patent foramen ovale (PFO), present in estimated 20–30% of the adult population [11, 12]. PFO is mostly asymptomatic and functions as a flap-like valve that transiently opens when right atrial pressure gets higher than the left atrial pressure during maneuvers like having bowel movement, coughing, or sneezing. However, a PFO can facilitate a new onset RLS in cases in which the pressure gradient across the atria is reversed (right atrial pressure > left atrial pressure). Conditions such as OSA, COPD, and other causes of pulmonary hypertension (PH) can lead to reversal of atrial pressure. The findings of normal right sided pressures with an accompanying RLS across a PFO appear to be a paradox. In our patient, RHC revealed normal right and left fillings pressures and normal pulmonary artery pressure and resistance, suggesting optimized COPD and OSA treatment, excluding reversal of atrial pressures as mechanism for RLS.

Hypoxemia secondary to RLS with normal pulmonary artery pressure has been extensively documented after right pneumonectomy whereas only few case reports have documented hypoxemia secondary to a RLS through a PFO in the presence of an elevated right hemidiaphragm, which our patient had due to viral pneumonia [11, 12]. We agree with the proposed mechanism by Zanchetta et al. for the paradoxical shunt based on an anatomical and embryological review of the flow dynamics from the inferior vena cava (IVC) into right atrium. Normally, blood enters the right atrium in upward and backward direction from IVC towards the foramen ovale (FO) to avoid collision with blood entering in downward and forward direction from the superior vena cava. In setting of right hemidiaphragm paralysis, blood flow from IVC is further directed towards the FO resulting in RLS through the PFO even in absence of reversed atrial pressures [10, 13]. Therefore, patient may not present with typical platypnea-orthodeoxia symptoms. Presence of exuberant Eustachian valve can also exacerbate the RLS via redirecting the flow towards the PFO in absence of reversed atrial pressures [14].

## 4. Conclusion

Right-to-left shunting through a PFO can occur due to multiple etiologies that either increase the right atrial pressure, reduce the left atrial pressure, or facilitate the IVC flow to be directed to the PFO. In some patients, PFO related RLS can lead to significant hypoxia and SOB. As in our case, high clinical suspicion and thorough evaluation helped reach the diagnosis of significant RLS via PFO in setting of right hemidiaphragm paralysis. Percutaneous PFO closure led to significant improvement in patient symptoms.

## Figures and Tables

**Table 1:** Literature review: reported cases of PFO and hemidiaphragm.

Patient #	Author, year	Age and gender	SOB duration	Hemidiaphragm diagnosis	Probable cause of paralysis	Side of hemidiaphragm	RAP (M/A/V) mmHg	PA Pressure (M/A/V) mmHg	PFO closure improving hypoxia	PaO2 correction on arterial blood gases on PFO closure
(1)	Murray et al., 1991 [2]	72, male	3 months	CXR, fluoroscopy	Idiopathic/Viral	Right	Normal	—	Yes	60 to 71 mmHg

(2)	Cordero et al., 1994 [3]	57, male	1 month	Chest X-ray, sniff test, EMG	Neurapraxia	Right	—	—	Not closed	37 to 78 mmHg

(3)	Ghamande et al., 2001 [4]	79, female	6 weeks	CT, sniff test	Postsurgical	Right	7/9/7	30/15/21	Yes	55 mmHg before closure

(4)	López Gastón et al., 2005 [5]	75, male	7 days	CXR, chest CT, fluoroscopy	L central line placement	Right	2	19.4	Yes	50 to 63 mmHg

(5)	Maholic and Lasorda, 2006 [6]	84, female	1 month	Chest X-ray, sniff test	Guillain Barre	Right	5, mean	22/5	Yes	48 to 67 mmHg

(6)	Perkins et al., 2008 [7]	73, female	2 weeks	Chest X-ray, Sniff Test	Idiopathic	Right	5, mean	14 mean	Yes	65 mmHg before closure

(7)	Fabris et al., 2015 [8]	66, female	Few weeks	Chest X-ray	Idiopathic	Right	3	12 mean	Yes	—

(8)	Sakagianni et al., 2012 [9]	72, female	3 weeks	Chest X-ray	After surgery	Right	14	36/19/24	Yes	Patient on vent at time of closure

(9)	Darchis et al., 2007 [10]	79, female	3 weeks	Chest X-ray	Liver mets and enlarged liver	Right	4	—	Yes	60 to 78 mmHg

(10)	Darchis et al., 2007 [10]	61, female	Acute	Chest X-ray	Postsurgical (phrenic nerve injury)	Right	5	—	Yes	61 to 84 mmHg

(11)	Darchis et al., 2007 [10]	84, female	3 weeks	Chest X-ray	Idiopathic	Right	—	—	Yes	49 to 74 mmHg
